# Supplementary material for: Seasonal Influenza Vaccine Effectiveness in Persons Aged 15–64 Years: A Systematic Review and Meta-Analysis
Source: Vaccines (Basel). 2023 Aug 4;11(8):1322. doi: 10.3390/vaccines11081322 (PMC10459161; doi:10.3390/vaccines11081322)
Supplement: Supplementary file 1 [file vaccines-11-01322-s001.zip › vaccines-2503190-supplementary.pdf]

Table S1. References of excluded studies

References	Reasons for exclusion
1. Zimmerman, R. K., Dauer, K., Clarke, L., Nowalk, M. P., Raviotta, J. M., & Balasubramani, G. K. (2023). Vaccine effectiveness of recombinant and standard dose influenza vaccines against outpatient illness during 2018-2019 and 2019-2020 calculated using a retrospective test-negative design. Human vaccines & immunotherapeutics, 19(1), 2177461. https://doi.org/10.1080/21645515.2023.2177461	Insufficient information to determine outcomes
2. Skowronski, D. M., Chuang, E. S., Sabaiduc, S., Kaweski, S. E., Kim, S., Dickinson, J. A., Olsha, R., Gubbay, J. B., Zelyas, N., Charest, H., Bastien, N., Jassem, A. N., & De Serres, G. (2023). Vaccine effectiveness estimates from an early-season influenza A(H3N2) epidemic, including unique genetic diversity with reassortment, Canada, 2022/23. Euro surveillance : bulletin Europeen sur les maladies transmissibles = European communicable disease bulletin, 28(5), 2300043. https://doi.org/10.2807/1560-7917.ES.2023.28.5.2300043	Not all strains
3. Panatto, D., Domnich, A., Chironna, M., Loconsole, D., Napoli, C., Torsello, A., Manini, I., Montomoli, E., Pariani, E., Castaldi, S., Orsi, A., Icardi, G., & On Behalf Of The It-Bive-Hosp Network Study Group (2022). Surveillance of Severe Acute Respiratory Infection and Influenza Vaccine Effectiveness among Hospitalized Italian Adults, 2021/22 Season. Vaccines, 11(1), 83. https://doi.org/10.3390/vaccines11010083	Adults not isolated
4. Stuurman, A. L., Levi, M., Beutels, P., Bricout, H., Descamps, A., Dos Santos, G., McGovern, I., Mira-Iglesias, A., Nauta, J., Torcel-Pagnon, L., Biccler, J., & DRIVE consortium (2023). Investigating confounding in network-based test-negative design influenza vaccine effectiveness studies-Experience from the DRIVE project. Influenza and other respiratory viruses, 17(1), e13087. https://doi.org/10.1111/irv.13087	Insufficient information to determine outcomes
5. Tenforde, M. W., Patel, M. M., Lewis, N. M., Adams, K., Gaglani, M., Steingrub, J. S., Shapiro, N. I., Duggal, A., Prekker, M. E., Peltan, I. D., Hager, D. N., Gong, M. N., Exline, M. C., Ginde, A. A., Mohr, N. M., Mallow, C., Martin, E. T., Talbot, H. K., Gibbs, K. W., Kwon, J. H., … Influenza and Other Viruses in the Acutely Ill (IVY) Network (2023). Vaccine Effectiveness Against Influenza A(H3N2)-Associated Hospitalized Illness: United States, 2022. Clinical infectious diseases : an official publication of the Infectious Diseases Society of America, 76(6), 1030–1037. https://doi.org/10.1093/cid/ciac869	Seasons not isolated
6. Hu, W., Sjoberg, P. A., DeMarcus, L. S., & Robbins, A. S. (2021). Influenza Vaccine Effectiveness Estimates among US Department of Defense Adult Beneficiaries over Four Consecutive Influenza Seasons: A Test-Negative Design Study with Different Control Groups. Vaccines, 10(1), 58. https://doi.org/10.3390/vaccines10010058	Seasons not isolated
7. Maltezou, H. C., Stavros, S., Asimakopoulos, G., Pergialiotis, V., Raftopoulos, V., Talias, M. A., Pavli, A., Daskalakis, G., Sindos, M., Koutroumanis, P., Theodora, M., Antsaklis, P., Kostis, E., Stratiki, E., Kossyvakis, A., Theodoridou, M., Mentis, A., Drakakis, P., Loutradis, D., & Rodolakis, A. (2022). Effectiveness of maternal vaccination with quadrivalent inactivated influenza vaccine in pregnant women and their infants in 2019-2020. Expert review of vaccines, 21(7), 983–992. https://doi.org/10.1080/14760584.2022.2013820	Not RCT or TND

8. Butler, C., Ellis, C., Folegatti, P. M., Swayze, H., Allen, J., Bussey, L., Bellamy, D., Lawrie, A., Eagling-Vose, E., Yu, L. M., Shanyinde, M., Mair, C., Flaxman, A., Ewer, K., Gilbert, S., Evans, T. G., & On Behalf Of The Invictus Investigators (2021). Efficacy and Safety of a Modified Vaccinia Ankara-NP+M1 Vaccine Combined with QIV in People Aged 65 and Older: A Randomised Controlled Clinical Trial (INVICTUS). Vaccines, 9(8), 851. https://doi.org/10.3390/vaccines9080851	Not a commercial vaccine
9. Martínez-Baz, I., Navascués, A., Casado, I., Aguinaga, A., Ezpeleta, C., & Castilla, J. (2021). Simple models to include influenza vaccination history when evaluating the effect of influenza vaccination. Euro surveillance : bulletin Europeen sur les maladies transmissibles = European communicable disease bulletin, 26(32), 2001099. https://doi.org/10.2807/1560-7917.ES.2021.26.32.2001099	Adults not isolated
10. Thervil, J. W., DeMarcus, L. S., Eick-Cost, A., Hu, Z., Myers, C. A., Hollis, E. M., Balansay-Ames, M. S., Ellis, K., & Christy, N. C. (2021). Department of Defense mid-season vaccine effectiveness estimates for the 2019-2020 influenza season. MSMR, 28(6), 16–19.	Insufficient information to determine outcomes
11. Ghamande, S., Shaver, C., Murthy, K., Raiyani, C., White, H. D., Lat, T., Arroliga, A. C., Wyatt, D., Talbot, H. K., Martin, E. T., Monto, A. S., Zimmerman, R. K., Middleton, D. B., Silveira, F. P., Ferdinands, J. M., Patel, M. M., & Gaglani, M. (2022). Vaccine Effectiveness Against Acute Respiratory Illness Hospitalizations for Influenza-Associated Pneumonia During the 2015-2016 to 2017-2018 Seasons: US Hospitalized Adult Influenza Vaccine Effectiveness Network (HAIVEN). Clinical infectious diseases : an official publication of the Infectious Diseases Society of America, 74(8), 1329–1337. https://doi.org/10.1093/cid/ciab654	Adults not isolated
12. Kuliese, M., Mickiene, A., Jancoriene, L., Zablockiene, B., Gefenaite, G., & Study Group (2021). Age-Specific Seasonal Influenza Vaccine Effectiveness against Different Influenza Subtypes in the Hospitalized Population in Lithuania during the 2015-2019 Influenza Seasons. Vaccines, 9(5), 455. https://doi.org/10.3390/vaccines9050455	Adults not isolated
13. Chan, Y. W., Wong, M. L., Kwok, F. Y., Au, A. K., Leung, E. C., & Chuang, S. K. (2021). The effect of seasonal influenza vaccine on medically-attended influenza and non-influenza respiratory viruses infections at primary care level, Hong Kong SAR, 2017/18 to 2019/20. Vaccine, 39(25), 3372–3378. https://doi.org/10.1016/j.vaccine.2021.04.059	Insufficient information to determine outcomes
14. de Lusignan, S., Hoang, U., Liyanage, H., Tripathy, M., Sherlock, J., Joy, M., Ferreira, F., Diez-Domingo, J., & Clark, T. (2021). Using Point of Care Testing to estimate influenza vaccine effectiveness in the English primary care sentinel surveillance network. PloS one, 16(3), e0248123. https://doi.org/10.1371/journal.pone.0248123	Adults not isolated
15. Balasubramani, G. K., Zimmerman, R. K., Eng, H., Lyons, J., Clarke, L., & Nowalk, M. P. (2021). Comparison of local influenza vaccine effectiveness using two methods. Vaccine, 39(8), 1283–1289. https://doi.org/10.1016/j.vaccine.2021.01.013	Adults not isolated
16. Hughes, K., Middleton, D. B., Nowalk, M. P., Balasubramani, G. K., Martin, E. T., Gaglani, M., Talbot, H. K., Patel, M. M., Ferdinands, J. M., Zimmerman, R. K., Silveira, F. P., & HAIVEN Study Investigators (2021). Effectiveness of Influenza Vaccine for Preventing Laboratory-Confirmed Influenza Hospitalizations in Immunocompromised Adults. Clinical infectious diseases : an official publication of the Infectious Diseases Society of America, 73(11), e4353–e4360. https://doi.org/10.1093/cid/ciaa1927	Adults not isolated

17. Tenforde, M. W., Talbot, H. K., Trabue, C. H., Gaglani, M., McNeal, T. M., Monto, A. S., Martin, E. T., Zimmerman, R. K., Silveira, F. P., Middleton, D. B., Olson, S. M., Garten Kondor, R. J., Barnes, J. R., Ferdinands, J. M., Patel, M. M., & Hospitalized Adult Influenza Vaccine Effectiveness Network (HAIVEN) Investigators (2021). Influenza Vaccine Effectiveness Against Hospitalization in the United States, 2019-2020. The Journal of infectious diseases, 224(5), 813–820. https://doi.org/10.1093/infdis/jiaa800	Insufficient information to determine outcomes
18. Tenforde, M. W., Kondor, R. J. G., Chung, J. R., Zimmerman, R. K., Nowalk, M. P., Jackson, M. L., Jackson, L. A., Monto, A. S., Martin, E. T., Belongia, E. A., McLean, H. Q., Gaglani, M., Rao, A., Kim, S. S., Stark, T. J., Barnes, J. R., Wentworth, D. E., Patel, M. M., & Flannery, B. (2021). Effect of Antigenic Drift on Influenza Vaccine Effectiveness in the United States-2019-2020. Clinical infectious diseases : an official publication of the Infectious Diseases Society of America, 73(11), e4244–e4250. https://doi.org/10.1093/cid/ciaa1884	Adults not isolated
19. Simpson, C. R., Lone, N. I., Kavanagh, K., Englishby, T., Robertson, C., McMenamin, J., Wissman, B. V., Vasileiou, E., Butler, C. C., Ritchie, L. D., Gunson, R., Schwarze, J., & Sheikh, A. (2020). Vaccine effectiveness of live attenuated and trivalent inactivated influenza vaccination in 2010/11 to 2015/16: the SIVE II record linkage study. Health technology assessment (Winchester, England), 24(67), 1–66. https://doi.org/10.3310/hta24670	Adults not isolated
20. Drori, Y., Pando, R., Sefty, H., Rosenberg, A., Mendelson, E., Keinan-Boker, L., Shohat, T., Mandelboim, M., Glatman-Freedman, A., & Israel Influenza Surveillance Network IISN (2020). Influenza vaccine effectiveness against laboratory-confirmed influenza in a vaccine-mismatched influenza B-dominant season. Vaccine, 38(52), 8387–8395. https://doi.org/10.1016/j.vaccine.2020.10.074	Insufficient information to determine outcomes
21. Tsuzuki, S., Ishikane, M., Matsunaga, N., Morioka, S., Yu, J., Inagaki, T., Yamamoto, M., & Ohmagari, N. (2021). Interim 2019/2020 Influenza Vaccine Effectiveness in Japan from October 2019 to January 2020. Japanese journal of infectious diseases, 74(3), 175–179. https://doi.org/10.7883/yoken.JJID.2020.177	Not RCT or TND
22. Smith, E. R., Fry, A. M., Hicks, L. A., Fleming-Dutra, K. E., Flannery, B., Ferdinands, J., Rolfes, M. A., Martin, E. T., Monto, A. S., Zimmerman, R. K., Nowalk, M. P., Jackson, M. L., McLean, H. Q., Olson, S. C., Gaglani, M., & Patel, M. M. (2020). Reducing Antibiotic Use in Ambulatory Care Through Influenza Vaccination. Clinical infectious diseases : an official publication of the Infectious Diseases Society of America, 71(11), e726–e734. https://doi.org/10.1093/cid/ciaa464	Adults not isolated
23. Balasubramani, G. K., Nowalk, M. P., Sax, T. M., Suyama, J., Bobyock, E., Rinaldo, C. R., Jr, Martin, E. T., Monto, A. S., Jackson, M. L., Gaglani, M. J., Flannery, B., Chung, J. R., & Zimmerman, R. K. (2020). Influenza vaccine effectiveness among outpatients in the US Influenza Vaccine Effectiveness Network by study site 2011-2016. Influenza and other respiratory viruses, 14(4), 380–390. https://doi.org/10.1111/irv.12741	Adults not isolated
24. Tenforde, M. W., Chung, J., Smith, E. R., Talbot, H. K., Trabue, C. H., Zimmerman, R. K., Silveira, F. P., Gaglani, M., Murthy, K., Monto, A. S., Martin, E. T., McLean, H. Q., Belongia, E. A., Jackson, L. A., Jackson, M. L., Ferdinands, J. M., Flannery, B., & Patel, M. M. (2021). Influenza Vaccine Effectiveness in Inpatient and Outpatient Settings in the United States, 2015-2018. Clinical infectious diseases : an official publication of the Infectious Diseases Society of America, 73(3), 386–392. https://doi.org/10.1093/cid/ciaa407	Insufficient information to determine outcomes

25. Glatman-Freedman, A., Pando, R., Sefty, H., Omer, I., Rosenberg, A., Drori, Y., Nemet, I., Mendelson, E., Keinan-Boker, L., Mandelboim, M., & Israeli Influenza Surveillance Network Iisn, F. T. (2020). Predominance of a Drifted Influenza A (H3N2) Clade and its Association with Age-specific Influenza Vaccine Effectiveness Variations, Influenza Season 2018-2019. Vaccines, 8(1), 78. https://doi.org/10.3390/vaccines8010078	Adults not isolated
26. Mouratidou, E., Lambrou, A., Andreopoulou, A., Gioula, G., Exindari, M., Kossyvakis, A., Pogka, V., Mentis, A., Georgakopoulou, T., & Lytras, T. (2020). Influenza vaccine effectiveness against hospitalization with laboratory-confirmed influenza in Greece: A pooled analysis across six seasons, 2013-2014 to 2018-2019. Vaccine, 38(12), 2715–2724. https://doi.org/10.1016/j.vaccine.2020.01.083	Adults not isolated
27. Castilla, J., Portillo, M. E., Casado, I., Pozo, F., Navascués, A., Adelantado, M., Gómez Ibáñez, C., Ezpeleta, C., Martínez-Baz, I., & Primary Health Care Sentinel Network and Network for Influenza Surveillance in Hospitals of Navarre (2020). Effectiveness of the current and prior influenza vaccinations in Northern Spain, 2018-2019. Vaccine, 38(8), 1925–1932. https://doi.org/10.1016/j.vaccine.2020.01.028	Seasons not isolated
28. Nation, M. L., Moss, R., Spittal, M. J., Kotsimbos, T., Kelly, P. M., & Cheng, A. C. (2021). Influenza Vaccine Effectiveness Against Influenza-Related Mortality in Australian Hospitalized Patients: A Propensity Score Analysis. Clinical infectious diseases : an official publication of the Infectious Diseases Society of America, 72(1), 99–107. https://doi.org/10.1093/cid/ciz1238	Adults not isolated
29. Skowronski, D. M., Sabaiduc, S., Leir, S., Rose, C., Zou, M., Murti, M., Dickinson, J. A., Olsha, R., Gubbay, J. B., Croxen, M. A., Charest, H., Bastien, N., Li, Y., Jassem, A., Krajden, M., & De Serres, G. (2019). Paradoxical clade- and age-specific vaccine effectiveness during the 2018/19 influenza A(H3N2) epidemic in Canada: potential imprint-regulated effect of vaccine (I-REV). Euro surveillance : bulletin Europeen sur les maladies transmissibles = European communicable disease bulletin, 24(46), 1900585. https://doi.org/10.2807/1560-7917.ES.2019.24.46.1900585	Not all strains
30. Vasileiou, E., Sheikh, A., Butler, C. C., Robertson, C., Kavanagh, K., Englishby, T., Lone, N. I., von Wissmann, B., McMenamin, J., Ritchie, L. D., Schwarze, J., Gunson, R., & Simpson, C. R. (2020). Seasonal Influenza Vaccine Effectiveness in People With Asthma: A National Test-Negative Design Case-Control Study. Clinical infectious diseases : an official publication of the Infectious Diseases Society of America, 71(7), e94–e104. https://doi.org/10.1093/cid/ciz1086	Adults not isolated
31. Pebody, R. G., Whitaker, H., Ellis, J., Andrews, N., Marques, D. F. P., Cottrell, S., Reynolds, A. J., Gunson, R., Thompson, C., Galiano, M., Lackenby, A., Robertson, C., O'Doherty, M. G., Owens, K., Yonova, I., Shepherd, S. J., Moore, C., Johnston, J., Donati, M., McMenamin, J., … Zambon, M. (2020). End of season influenza vaccine effectiveness in primary care in adults and children in the United Kingdom in 2018/19. Vaccine, 38(3), 489–497. https://doi.org/10.1016/j.vaccine.2019.10.071	Adults not isolated
32. Colucci, M. E., Veronesi, L., Bracchi, M. T., Zoni, R., Caruso, L., Capobianco, E., Rossi, D., Bizzarro, A., Cantarelli, A., & Affanni, P. (2019). On field vaccine effectiveness in three periods of 2018/2019 influenza season in Emilia-Romagna Region. Acta bio-medica : Atenei Parmensis, 90(9-S), 21–27. https://doi.org/10.23750/abm.v90i9-S.8699	Adults not isolated

33. Castillejos, M., Cabello-Gutiérrez, C., Alberto Choreño-Parra, J., Hernández, V., Romo, J., Hernández-Sánchez, F., Martínez, D., Hernández, A., Jiménez-Álvarez, L., Hernández-Cardenas, C. M., Becerril-Vargas, E., Martínez-Orozco, J. A., Luis Sandoval-Gutiérrez, J., Guadarrama, C., Olvera-Masetto, E., Alfaro-Ramos, L., Cruz-Lagunas, A., Ramírez, G., Márquez, E., Pimentel, L., … Zúñiga, J. (2019). High performance of rapid influenza diagnostic test and variable effectiveness of influenza vaccines in Mexico. International journal of infectious diseases : IJID : official publication of the International Society for Infectious Diseases, 89, 87–95. https://doi.org/10.1016/j.ijid.2019.08.029	Adults not isolated
34. Ng, Y., Nandar, K., Chua, L. A. V., Mak, T. M., Foo, K., Muhammad, I. R., Low, C. K. K., Ma, S., Ooi, S. P., Lin, R. T. P., James, L., & Lee, V. J. M. (2019). Evaluating the effectiveness of the influenza vaccine during respiratory outbreaks in Singapore's long term care facilities, 2017. Vaccine, 37(29), 3925–3931. https://doi.org/10.1016/j.vaccine.2019.03.054	Adults not isolated
35. Puig-Barberà, J., Mira-Iglesias, A., Burtseva, E., Cowling, B. J., Serhat, U., Ruiz-Palacios, G. M., Launay, O., Kyncl, J., Koul, P., Siqueira, M. M., Sominina, A., & Global Influenza Hospital Surveillance Network (2019). Influenza epidemiology and influenza vaccine effectiveness during the 2015-2016 season: results from the Global Influenza Hospital Surveillance Network. BMC infectious diseases, 19(1), 415. https://doi.org/10.1186/s12879-019-4017-0	Adults not isolated
36. Coleman, R., Eick-Cost, A. A., Hawksworth, A. W., Hu, Z., Lynch, L., Myers, C. A., DeMarcus, L., & Federinko, S. (2018). Department of Defense end-of-season influenza vaccine effectiveness estimates for the 2017-2018 season. MSMR, 25(10), 16–20.	Insufficient information to determine outcomes
37. Baselga-Moreno, V., Trushakova, S., McNeil, S., Sominina, A., Nunes, M. C., Draganescu, A., Unal, S., Koul, P., Kyncl, J., Zhang, T., Kuatbayeva, A., Ben-Salah, A., Burtseva, E., Puig-Barberà, J., Díez-Domingo, J., & Global Influenza Hospital Surveillance Network (GIHSN) (2019). Influenza epidemiology and influenza vaccine effectiveness during the 2016-2017 season in the Global Influenza Hospital Surveillance Network (GIHSN). BMC public health, 19(1), 487. https://doi.org/10.1186/s12889-019-6713-5	Adults not isolated
38. Rolfes, M. A., Flannery, B., Chung, J. R., O'Halloran, A., Garg, S., Belongia, E. A., Gaglani, M., Zimmerman, R. K., Jackson, M. L., Monto, A. S., Alden, N. B., Anderson, E., Bennett, N. M., Billing, L., Eckel, S., Kirley, P. D., Lynfield, R., Monroe, M. L., Spencer, M., Spina, N., … US Influenza Vaccine Effectiveness (Flu VE) Network, the Influenza Hospitalization Surveillance Network, and the Assessment Branch, Immunization Services Division, Centers for Disease Control and Prevention (2019). Effects of Influenza Vaccination in the United States During the 2017-2018 Influenza Season. Clinical infectious diseases : an official publication of the Infectious Diseases Society of America, 69(11), 1845–1853. https://doi.org/10.1093/cid/ciz075	Insufficient information to determine outcomes
39. Ferdinands, J. M., Gaglani, M., Martin, E. T., Middleton, D., Monto, A. S., Murthy, K., Silveira, F. P., Talbot, H. K., Zimmerman, R., Alyanak, E., Strickland, C., Spencer, S., Fry, A. M., & HAIVEN Study Investigators (2019). Prevention of Influenza Hospitalization Among Adults in the United States, 2015-2016: Results From the US Hospitalized Adult Influenza Vaccine Effectiveness Network (HAIVEN). The Journal of infectious diseases, 220(8), 1265–1275. https://doi.org/10.1093/infdis/jiy723	Adults not isolated

40. Shang, M., Chung, J. R., Jackson, M. L., Jackson, L. A., Monto, A. S., Martin, E. T., Belongia, E. A., McLean, H. Q., Gaglani, M., Murthy, K., Zimmerman, R. K., Nowalk, M. P., Fry, A. M., & Flannery, B. (2018). Influenza vaccine effectiveness among patients with high-risk medical conditions in the United States, 2012-2016. Vaccine, 36(52), 8047–8053. https://doi.org/10.1016/j.vaccine.2018.10.093	Adults not isolated
41. Tam, Y. H., Ng, T. W. Y., Chu, D. K. W., Fang, V. J., Cowling, B. J., Malik Peiris, J. S., & Ip, D. K. M. (2018). The effectiveness of influenza vaccination against medically-attended illnesses in Hong Kong across three years with different degrees of vaccine match, 2014-17. Vaccine, 36(41), 6117–6123. https://doi.org/10.1016/j.vaccine.2018.08.075	Seasons not isolated
42. Thompson, M. G., Pierse, N., Sue Huang, Q., Prasad, N., Duque, J., Claire Newbern, E., Baker, M. G., Turner, N., McArthur, C., & SHIVERS investigation team (2018). Influenza vaccine effectiveness in preventing influenza-associated intensive care admissions and attenuating severe disease among adults in New Zealand 2012-2015. Vaccine, 36(39), 5916–5925. https://doi.org/10.1016/j.vaccine.2018.07.028	Adults not isolated
43. Andrew, M. K., Shinde, V., Hatchette, T., Ambrose, A., Boivin, G., Bowie, W., Chit, A., Dos Santos, G., ElSherif, M., Green, K., Haguinet, F., Halperin, S. A., Ibarguchi, B., Johnstone, J., Katz, K., Langley, J. M., LeBlanc, J., Loeb, M., MacKinnon-Cameron, D., McCarthy, A., … Public Health Agency of Canada/Canadian Institutes of Health Research Influenza Research Network (PCIRN) Serious Outcomes Surveillance Network and the Toronto Invasive Bacterial Diseases Network (TIBDN) (2017). Influenza vaccine effectiveness against influenza-related hospitalization during a season with mixed outbreaks of four influenza viruses: a test-negative case-control study in adults in Canada. BMC infectious diseases, 17(1), 805. https://doi.org/10.1186/s12879-017-2905-8	Adults not isolated
44. Puig-Barberà, J., Mira-Iglesias, A., Tortajada-Girbés, M., López-Labrador, F. X., Librero-López, J., Díez-Domingo, J., Carballido-Fernández, M., Carratalá-Munuera, C., Correcher-Medina, P., Gil-Guillén, V., Limón-Ramírez, R., Mollar-Maseres, J., Otero-Reigada, M. C., Schwarz, H., & Valencia Hospital Network for the Study of Influenza and other Respiratory Viruses (VAHNSI, Spain) (2017). Waning protection of influenza vaccination during four influenza seasons, 2011/2012 to 2014/2015. Vaccine, 35(43), 5799–5807. https://doi.org/10.1016/j.vaccine.2017.09.035	Insufficient information to determine outcomes
45. Hekimoğlu, C. H., Emek, M., Avcı, E., Topal, S., Demiröz, M., & Ergör, G. (2018). Seasonal Influenza Vaccine Effectiveness in Preventing Laboratory Confirmed Influenza in 2014-2015 Season in Turkey: A Test-Negative Case Control Study. Balkan medical journal, 35(1), 77–83. https://doi.org/10.4274/balkanmedj.2017.0487	Adults not isolated
46. Gherasim, A., Martínez-Baz, I., Castilla, J., Pozo, F., Larrauri, A., & cycEVA working group (2017). Effect of previous and current vaccination against influenza A(H1N1)pdm09, A(H3N2), and B during the post-pandemic period 2010-2016 in Spain. PloS one, 12(6), e0179160. https://doi.org/10.1371/journal.pone.0179160	Insufficient information to determine outcomes
47. van Doorn, E., Darvishian, M., Dijkstra, F., Bijlsma, M. J., Donker, G. A., de Lange, M. M. A., Cadenau, L. M., Hak, E., & Meijer, A. (2017). Effectiviteit van influenzavaccinatie in Nederland [Effectiveness of influenza vaccine in the Netherlands: predominant circulating virus type and vaccine match are important conditions]. Nederlands tijdschrift voor geneeskunde, 161, D1648.	Not English

48. Noh, J. Y., Lim, S., Song, J. Y., Choi, W. S., Jeong, H. W., Heo, J. Y., Lee, J., Seo, Y. B., Lee, J. S., Wie, S. H., Kim, Y. K., Park, K. H., Jung, S. I., Kim, S. W., Lee, S. H., Lee, H. S., Yoon, Y. H., Cheong, H. J., & Kim, W. J. (2017). Interim estimates of the effectiveness of the influenza vaccine against A(H3N2) influenza in adults in South Korea, 2016-2017 season. PloS one, 12(5), e0178010. https://doi.org/10.1371/journal.pone.0178010	Not English
49. Zhang, Y., Wu, P., Feng, L., Yang, P., Pan, Y., Feng, S., Qin, Y., Zheng, J., Puig-Barberà, J., Muscatello, D., MacIntyre, R., Cowling, B. J., Yu, H., & Wang, Q. (2017). Influenza vaccine effectiveness against influenza-associated hospitalization in 2015/16 season, Beijing, China. Vaccine, 35(23), 3129–3134. https://doi.org/10.1016/j.vaccine.2017.03.084	Adults not isolated
50. Martínez-Baz, I., Casado, I., Navascués, A., Díaz-González, J., Aguinaga, A., Barrado, L., Delfrade, J., Ezpeleta, C., & Castilla, J. (2017). Effect of Repeated Vaccination With the Same Vaccine Component Against 2009 Pandemic Influenza A(H1N1) Virus. The Journal of infectious diseases, 215(6), 847–855. https://doi.org/10.1093/infdis/jix055	Adults not isolated
51. van Doorn, E., Darvishian, M., Dijkstra, F., Donker, G. A., Overduin, P., Meijer, A., & Hak, E. (2017). Influenza vaccine effectiveness estimates in the Dutch population from 2003 to 2014: The test-negative design case-control study with different control groups. Vaccine, 35(21), 2831–2839. https://doi.org/10.1016/j.vaccine.2017.04.012	Insufficient information to determine outcomes
52. Cheng, A. C., Macartney, K. K., Waterer, G. W., Kotsimbos, T., Kelly, P. M., Blyth, C. C., & Influenza Complications Alert Network (FluCAN) Investigators (2017). Repeated Vaccination Does Not Appear to Impact Upon Influenza Vaccine Effectiveness Against Hospitalization With Confirmed Influenza. Clinical infectious diseases : an official publication of the Infectious Diseases Society of America, 64(11), 1564–1572. https://doi.org/10.1093/cid/cix209	Seasons not isolated
53. Skowronski, D. M., Chambers, C., Sabaiduc, S., Dickinson, J. A., Winter, A. L., De Serres, G., Drews, S. J., Jassem, A., Gubbay, J. B., Charest, H., Balshaw, R., Bastien, N., Li, Y., & Krajden, M. (2017). Interim estimates of 2016/17 vaccine effectiveness against influenza A(H3N2), Canada, January 2017. Euro surveillance : bulletin Europeen sur les maladies transmissibles = European communicable disease bulletin, 22(6), 30460. https://doi.org/10.2807/1560-7917.ES.2017.22.6.30460	Not all strains
54. Skowronski, D. M., Chambers, C., De Serres, G., Sabaiduc, S., Winter, A. L., Dickinson, J. A., Gubbay, J. B., Fonseca, K., Drews, S. J., Charest, H., Martineau, C., Krajden, M., Petric, M., Bastien, N., Li, Y., & Smith, D. J. (2017). Serial Vaccination and the Antigenic Distance Hypothesis: Effects on Influenza Vaccine Effectiveness During A(H3N2) Epidemics in Canada, 2010-2011 to 2014-2015. The Journal of infectious diseases, 215(7), 1059–1099. https://doi.org/10.1093/infdis/jix074	Insufficient information to determine outcomes
55. Darvishian, M., Dijkstra, F., van Doorn, E., Bijlsma, M. J., Donker, G. A., de Lange, M. M., Cadenau, L. M., Hak, E., & Meijer, A. (2017). Influenza Vaccine Effectiveness in the Netherlands from 2003/2004 through 2013/2014: The Importance of Circulating Influenza Virus Types and Subtypes. PloS one, 12(1), e0169528. https://doi.org/10.1371/journal.pone.0169528	Seasons not isolated
56. Saito, N., Komori, K., Suzuki, M., Morimoto, K., Kishikawa, T., Yasaka, T., & Ariyoshi, K. (2017). Negative impact of prior influenza vaccination on current influenza vaccination among people infected and not infected in prior season: A test-negative case-control study in Japan. Vaccine, 35(4), 687–693. https://doi.org/10.1016/j.vaccine.2016.11.024	Insufficient information to determine outcomes

57. Seki, Y., Oonishi, H., Onose, A., & Sugaya, N. (2016). Kansenshogaku zasshi. The Journal of the Japanese Association for Infectious Diseases, 90(4), 486–492.	Not English
58. Flannery, B., Zimmerman, R. K., Gubareva, L. V., Garten, R. J., Chung, J. R., Nowalk, M. P., Jackson, M. L., Jackson, L. A., Monto, A. S., Ohmit, S. E., Belongia, E. A., McLean, H. Q., Gaglani, M., Piedra, P. A., Mishin, V. P., Chesnokov, A. P., Spencer, S., Thaker, S. N., Barnes, J. R., Foust, A., … Fry, A. M. (2016). Enhanced Genetic Characterization of Influenza A(H3N2) Viruses and Vaccine Effectiveness by Genetic Group, 2014-2015. The Journal of infectious diseases, 214(7), 1010–1019. https://doi.org/10.1093/infdis/jiw181	Insufficient information to determine outcomes
59. Qin, Y., Zhang, Y., Wu, P., Feng, S., Zheng, J., Yang, P., Pan, Y., Wang, Q., Feng, L., Pang, X., Puig-Barberà, J., Yu, H., & Cowling, B. J. (2016). Influenza vaccine effectiveness in preventing hospitalization among Beijing residents in China, 2013-15. Vaccine, 34(20), 2329–2333. https://doi.org/10.1016/j.vaccine.2016.03.068	Seasons not isolated
60. Chambers, C., Skowronski, D. M., Sabaiduc, S., Winter, A. L., Dickinson, J. A., De Serres, G., Gubbay, J. B., Drews, S. J., Martineau, C., Eshaghi, A., Krajden, M., Bastien, N., & Li, Y. (2016). Interim estimates of 2015/16 vaccine effectiveness against influenza A(H1N1)pdm09, Canada, February 2016. Euro surveillance : bulletin Europeen sur les maladies transmissibles = European communicable disease bulletin, 21(11), 30168. https://doi.org/10.2807/1560-7917.ES.2016.21.11.30168	Adults not isolated
61. Talbot, H. K., Nian, H., Chen, Q., Zhu, Y., Edwards, K. M., & Griffin, M. R. (2016). Evaluating the case-positive, control test-negative study design for influenza vaccine effectiveness for the frailty bias. Vaccine, 34(15), 1806–1809. https://doi.org/10.1016/j.vaccine.2016.02.037	Adults not isolated
62. Castilla, J., Navascués, A., Fernández-Alonso, M., Reina, G., Pozo, F., Casado, I., Guevara, M., Martínez-Baz, I., Barricarte, A., Ezpeleta, C., Primary Health Care Sentinel Network, & Network for Influenza Surveillance in Hospitals of Navarra (2016). Effectiveness of subunit influenza vaccination in the 2014-2015 season and residual effect of split vaccination in previous seasons. Vaccine, 34(11), 1350–1357. https://doi.org/10.1016/j.vaccine.2016.01.054	Insufficient information to determine outcomes
63. Lane, C. R., Carville, K. S., Pierse, N., & Kelly, H. A. (2016). Seasonal influenza vaccine effectiveness estimates: Development of a parsimonious case test negative model using a causal approach. Vaccine, 34(8), 1070–1076. https://doi.org/10.1016/j.vaccine.2016.01.002	Insufficient information to determine outcomes
64. Gaglani, M., Pruszynski, J., Murthy, K., Clipper, L., Robertson, A., Reis, M., Chung, J. R., Piedra, P. A., Avadhanula, V., Nowalk, M. P., Zimmerman, R. K., Jackson, M. L., Jackson, L. A., Petrie, J. G., Ohmit, S. E., Monto, A. S., McLean, H. Q., Belongia, E. A., Fry, A. M., & Flannery, B. (2016). Influenza Vaccine Effectiveness Against 2009 Pandemic Influenza A(H1N1) Virus Differed by Vaccine Type During 2013-2014 in the United States. The Journal of infectious diseases, 213(10), 1546–1556. https://doi.org/10.1093/infdis/jiv577	Insufficient information to determine outcomes
65. Pierse, N., Kelly, H., Thompson, M. G., Bissielo, A., Radke, S., Huang, Q. S., Baker, M. G., Turner, N., & SHIVERS investigation team (2016). Influenza vaccine effectiveness for hospital and community patients using control groups with and without non-influenza respiratory viruses detected, Auckland, New Zealand 2014. Vaccine, 34(4), 503–509. https://doi.org/10.1016/j.vaccine.2015.11.073	Insufficient information to determine outcomes

66. Pebody, R., Warburton, F., Andrews, N., Ellis, J., von Wissmann, B., Robertson, C., Yonova, I., Cottrell, S., Gallagher, N., Green, H., Thompson, C., Galiano, M., Marques, D., Gunson, R., Reynolds, A., Moore, C., Mullett, D., Pathirannehelage, S., Donati, M., Johnston, J., … Zambon, M. (2015). Effectiveness of seasonal influenza vaccine in preventing laboratory-confirmed influenza in primary care in the United Kingdom: 2014/15 end of season results. Euro surveillance : bulletin Europeen sur les maladies transmissibles = European communicable disease bulletin, 20(36), 10.2807/1560-7917.ES.2015.20.36.30013. https://doi.org/10.2807/1560-7917.ES.2015.20.36.30013	Insufficient information to determine outcomes
67. Jimenez-Jorge, S., de Mateo, S., Delgado-Sanz, C., Pozo, F., Casas, I., Garcia-Cenoz, M., Castilla, J., Rodriguez, C., Vega, T., Quinones, C., Martinez, E., Vanrell, J. M., Gimenez, J., Castrillejo, D., Altzibar, J. M., Carril, F., Ramos, J. M., Serrano, M. C., Martinez, A., Torner, N., … Spanish Influenza Sentinel Surveillance System (2015). Estimating influenza vaccine effectiveness in Spain using sentinel surveillance data. Euro surveillance : bulletin Europeen sur les maladies transmissibles = European communicable disease bulletin, 20(28), 21187. https://doi.org/10.2807/1560-7917.es2015.20.28.21187	Not all strains
68. Martínez-Baz, I., Navascués, A., Pozo, F., Chamorro, J., Albeniz, E., Casado, I., Reina, G., Cenoz, M. G., Ezpeleta, C., Castilla, J., & Primary Health Care Sentinel Network and Network for Influenza Surveillance in Hospitals of Navarra (2015). Influenza vaccine effectiveness in preventing inpatient and outpatient cases in a season dominated by vaccine-matched influenza B virus. Human vaccines & immunotherapeutics, 11(7), 1626–1633. https://doi.org/10.1080/21645515.2015.1038002	Adults not isolated
69. Valenciano, M., Kissling, E., Reuss, A., Jiménez-Jorge, S., Horváth, J. K., Donnell, J. M., Pitigoi, D., Machado, A., Pozo, F., & I-MOVE Multicentre Case Control Study Team (2015). The European I-MOVE Multicentre 2013-2014 Case-Control Study. Homogeneous moderate influenza vaccine effectiveness against A(H1N1)pdm09 and heterogenous results by country against A(H3N2). Vaccine, 33(24), 2813–2822. https://doi.org/10.1016/j.vaccine.2015.04.012	Not all strains
70. Jiménez-Jorge, S., Pozo, F., Larrauri, A., & cycEVA Study Team (2015). Interim influenza vaccine effectiveness: A good proxy for final estimates in Spain in the seasons 2010-2014. Vaccine, 33(29), 3276–3280. https://doi.org/10.1016/j.vaccine.2015.03.051	Adults not isolated
71. Simpson, C. R., Lone, N. I., Kavanagh, K., Ritchie, L. D., Robertson, C., Sheikh, A., & McMenamin, J. (2015). Trivalent inactivated seasonal influenza vaccine effectiveness for the prevention of laboratory-confirmed influenza in a Scottish population 2000 to 2009. Euro surveillance : bulletin Europeen sur les maladies transmissibles = European communicable disease bulletin, 20(8), 21043. https://doi.org/10.2807/1560-7917.es2015.20.8.21043	Adults not isolated
72. Lytras, T., Kossyvakis, A., Melidou, A., Exindari, M., Gioula, G., Pogka, V., Malisiovas, N., & Mentis, A. (2015). Influenza vaccine effectiveness against laboratory confirmed influenza in Greece during the 2013-2014 season: a test-negative study. Vaccine, 33(2), 367–373. https://doi.org/10.1016/j.vaccine.2014.11.005	Adults not isolated
73. Carville, K. S., Grant, K. A., Sullivan, S. G., Fielding, J. E., Lane, C. R., Franklin, L., Druce, J., & Kelly, H. A. (2015). Understanding influenza vaccine protection in the community: an assessment of the 2013 influenza season in Victoria, Australia. Vaccine, 33(2), 341–345. https://doi.org/10.1016/j.vaccine.2014.11.019	Adults not isolated

74. Puig-Barberà, J., García-de-Lomas, J., Díez-Domingo, J., Arnedo-Pena, A., Ruiz-García, M., Limón-Ramírez, R., Pérez-Vilar, S., Micó-Esparza, J. L., Tortajada-Girbés, M., Carratalá-Munuera, C., Larrea-González, R., Beltrán-Garrido, J. M., Otero-Reigada, M.delC., Mollar-Maseres, J., Correcher-Medina, P., Schwarz-Chavarri, G., Gil-Guillén, V., & Valencia Hospital Network for the Study of Influenza and Respiratory Virus Disease (2014). Influenza vaccine effectiveness in preventing influenza A(H3N2)-related hospitalizations in adults targeted for vaccination by type of vaccine: a hospital-based test-negative study, 2011-2012 A(H3N2) predominant influenza season, Valencia, Spain. PloS one, 9(11), e112294. https://doi.org/10.1371/journal.pone.0112294	Not all strains
75. Cheng, A. C., Dwyer, D. E., Holmes, M., Irving, L. B., Brown, S. G., Waterer, G. W., Korman, T. M., Hunter, C., Hewagama, S., Friedman, N. D., Wark, P. A., Simpson, G., Upham, J. W., Bowler, S. D., Senenayake, S. N., Kotsimbos, T. C., & Kelly, P. M. (2014). Influenza epidemiology, vaccine coverage and vaccine effectiveness in sentinel Australian hospitals in 2013: the Influenza Complications Alert Network. Communicable diseases intelligence quarterly report, 38(2), E143–E149.	Adults not isolated
76. Turner, N., Pierse, N., Bissielo, A., Huang, Q., Radke, S., Baker, M., Widdowson, M., Kelly, H., & SHIVERS investigation team (2014). Effectiveness of seasonal trivalent inactivated influenza vaccine in preventing influenza hospitalisations and primary care visits in Auckland, New Zealand, in 2013. Euro surveillance : bulletin Europeen sur les maladies transmissibles = European communicable disease bulletin, 19(34), 20884.	Adults not isolated
77. Andrews, N., McMenamin, J., Durnall, H., Ellis, J., Lackenby, A., Robertson, C., von Wissmann, B., Cottrell, S., Smyth, B., Moore, C., Gunson, R., Zambon, M., Fleming, D., & Pebody, R. (2014). Effectiveness of trivalent seasonal influenza vaccine in preventing laboratory-confirmed influenza in primary care in the United Kingdom: 2012/13 end of season results. Euro surveillance : bulletin Europeen sur les maladies transmissibles = European communicable disease bulletin, 19(27), 5–13.	Adults not isolated
78. Savulescu, C., Jiménez-Jorge, S., Delgado-Sanz, C., de Mateo, S., Pozo, F., Casas, I., Larrauri, A., & Spanish Influenza Surveillance System (2014). Higher vaccine effectiveness in seasons with predominant circulation of seasonal influenza A(H1N1) than in A(H3N2) seasons: test-negative case-control studies using surveillance data, Spain, 2003-2011. Vaccine, 32(35), 4404–4411. https://doi.org/10.1016/j.vaccine.2014.06.063	Adults not isolated
79. Nunes, B., Machado, A., Guiomar, R., Pechirra, P., Conde, P., Cristovão, P., & Falcão, I. (2014). Estimates of 2012/13 influenza vaccine effectiveness using the case test-negative control design with different influenza negative control groups. Vaccine, 32(35), 4443–4449. https://doi.org/10.1016/j.vaccine.2014.06.053	Adults not isolated
80. Puig-Barberà, J., Natividad-Sancho, A., Launay, O., Burtseva, E., Ciblak, M. A., Tormos, A., Buigues-Vila, A., Martínez-Úbeda, S., Sominina, A., & GIHSN Group (2014). 2012-2013 Seasonal influenza vaccine effectiveness against influenza hospitalizations: results from the global influenza hospital surveillance network. PloS one, 9(6), e100497. https://doi.org/10.1371/journal.pone.0100497	Insufficient information to determine outcomes
81. Ho, H. P., Zhao, X., Pang, J., Chen, M. I., Lee, V. J., Ang, L. W., Lin, R. V., Gao, C. Q., Hsu, L. Y., & Cook, A. R. (2014). Effectiveness of seasonal influenza vaccinations against laboratory-confirmed influenza-associated infections among Singapore military personnel in 2010-2013. Influenza and other respiratory viruses, 8(5), 557–566. https://doi.org/10.1111/irv.12256	Adults not isolated

82. Turner, N., Pierse, N., Bissielo, A., Huang, Q. S., Baker, M. G., Widdowson, M. A., Kelly, H., & SHIVERS investigation team (2014). The effectiveness of seasonal trivalent inactivated influenza vaccine in preventing laboratory confirmed influenza hospitalisations in Auckland, New Zealand in 2012. Vaccine, 32(29), 3687–3693. https://doi.org/10.1016/j.vaccine.2014.04.013	Adults not isolated
83. McNeil, S., Shinde, V., Andrew, M., Hatchette, T., Leblanc, J., Ambrose, A., Boivin, G., Bowie, W., Diaz-Mitoma, F., Elsherif, M., Green, K., Haguinet, F., Halperin, S., Ibarguchi, B., Katz, K., Langley, J., Lagace-Wiens, P., Light, B., Loeb, M., McElhaney, J., … Toronto Invasive Bacterial Diseases Network (TIBDN) (2014). Interim estimates of 2013/14 influenza clinical severity and vaccine effectiveness in the prevention of laboratory-confirmed influenza-related hospitalisation, Canada, February 2014. Euro surveillance : bulletin Europeen sur les maladies transmissibles = European communicable disease bulletin, 19(9), 20729. https://doi.org/10.2807/1560-7917.es2014.19.9.20729	Adults not isolated
84. Suzuki, M., Minh, leN., Yoshimine, H., Inoue, K., Yoshida, L. M., Morimoto, K., & Ariyoshi, K. (2014). Vaccine effectiveness against medically attended laboratory-confirmed influenza in Japan, 2011-2012 Season. PloS one, 9(2), e88813. https://doi.org/10.1371/journal.pone.0088813	Adults not isolated
85. Dawood, F. S., Prapasiri, P., Areerat, P., Ruayajin, A., Chittaganpitch, M., Muangchana, C., Baggett, H. C., & Olsen, S. J. (2014). Effectiveness of the 2010 and 2011 Southern Hemisphere trivalent inactivated influenza vaccines against hospitalization with influenza-associated acute respiratory infection among Thai adults aged ≥ 50 years. Influenza and other respiratory viruses, 8(4), 463–468. https://doi.org/10.1111/irv.12233	Adults not isolated
86. Kulkarni, P. S., Agarkhedkar, S., Lalwani, S., Bavdekar, A. R., Jog, S., Raut, S. K., Parulekar, V., Agarkhedkar, S. S., Palkar, S., & Mangrule, S. (2014). Effectiveness of an Indian-made attenuated influenza A(H1N1)pdm 2009 vaccine: a case control study. Human vaccines & immunotherapeutics, 10(3), 566–571. https://doi.org/10.4161/hv.27490	Adults not isolated
87. Gefenaite, G., Rahamat-Langendoen, J., Ambrozaitis, A., Mickiene, A., Jancoriene, L., Kuliese, M., Velyvyte, D., Niesters, H., Stolk, R. P., Zagminas, K., & Hak, E. (2014). Seasonal influenza vaccine effectiveness against influenza in 2012-2013: a hospital-based case-control study in Lithuania. Vaccine, 32(7), 857–863. https://doi.org/10.1016/j.vaccine.2013.12.021	Adults not isolated
88. Cheng, A. C., Holmes, M., Irving, L. B., Brown, S. G., Waterer, G. W., Korman, T. M., Friedman, N. D., Senanayake, S., Dwyer, D. E., Brady, S., Simpson, G., Wood-Baker, R., Upham, J., Paterson, D., Jenkins, C., Wark, P., Kelly, P. M., & Kotsimbos, T. (2013). Influenza vaccine effectiveness against hospitalisation with confirmed influenza in the 2010-11 seasons: a test-negative observational study. PloS one, 8(7), e68760. https://doi.org/10.1371/journal.pone.0068760	Insufficient information to determine outcomes
89. Martínez-Baz, I., Martínez-Artola, V., Reina, G., Guevara, M., Cenoz, M. G., Morán, J., Irisarri, F., Arriazu, M., Albeniz, E., Castilla, J., & Primary Health Care Sentinel Network of Navarre (2013). Effectiveness of the trivalent influenza vaccine in Navarre, Spain, 2010-2011: a population-based test-negative case-control study. BMC public health, 13, 191. https://doi.org/10.1186/1471-2458-13-191	Adults not isolated
90. Talbot, H. K., Zhu, Y., Chen, Q., Williams, J. V., Thompson, M. G., & Griffin, M. R. (2013). Effectiveness of influenza vaccine for preventing laboratory-confirmed influenza hospitalizations in adults, 2011-2012 influenza season. Clinical infectious diseases : an official publication of the Infectious Diseases Society of America, 56(12), 1774–1777. https://doi.org/10.1093/cid/cit124	Adults not isolated

91. Valenciano, M., Kissling, E., & I-MOVE Case-Control Study Team (2013). Early estimates of seasonal influenza vaccine effectiveness in Europe: results from the I-MOVE multicentre case-control study, 2012/13. Euro surveillance : bulletin Europeen sur les maladies transmissibles = European communicable disease bulletin, 18(7), 3.	Adults not isolated
92. Castilla, J., Martinez-Baz, I., Martinez-Artola, V., Fernandez-Alonso, M., Reina, G., Guevara, M., Garcia Cenoz, M., Elia, F., Alvarez, N., Barricarte, A., & Ezpeleta, C. (2013). Early estimates of influenza vaccine effectiveness in Navarre, Spain: 2012/13 mid-season analysis. Euro surveillance : bulletin Europeen sur les maladies transmissibles = European communicable disease bulletin, 18(7), 2.	Adults not isolated
93. Pebody, R., Andrews, N., McMenamin, J., Durnall, H., Ellis, J., Thompson, C. I., Robertson, C., Cottrell, S., Smyth, B., Zambon, M., Moore, C., Fleming, D. M., & Watson, J. M. (2013). Vaccine effectiveness of 2011/12 trivalent seasonal influenza vaccine in preventing laboratory-confirmed influenza in primary care in the United Kingdom: evidence of waning intra-seasonal protection. Euro surveillance : bulletin Europeen sur les maladies transmissibles = European communicable disease bulletin, 18(5), 20389. https://doi.org/10.2807/ese.18.05.20389-en	Adults not isolated
94. Skowronski, D. M., Janjua, N. Z., De Serres, G., Dickinson, J. A., Winter, A. L., Mahmud, S. M., Sabaiduc, S., Gubbay, J. B., Charest, H., Petric, M., Fonseca, K., Van Caeseele, P., Kwindt, T. L., Krajden, M., Eshaghi, A., & Li, Y. (2013). Interim estimates of influenza vaccine effectiveness in 2012/13 from Canada's sentinel surveillance network, January 2013. Euro surveillance : bulletin Europeen sur les maladies transmissibles = European communicable disease bulletin, 18(5), 20394. https://doi.org/10.2807/ese.18.05.20394-en	Adults not isolated
95. MacIntosh, V. H., Tastad, K. J., & Eick-Cost, A. A. (2013). Mid-season influenza vaccine effectiveness 2011-2012: a Department of Defense Global, Laboratory-based, Influenza Surveillance System case-control study estimate. Vaccine, 31(13), 1651–1655. https://doi.org/10.1016/j.vaccine.2013.01.022	Insufficient information to determine outcomes
96. Bateman, A. C., Kieke, B. A., Irving, S. A., Meece, J. K., Shay, D. K., & Belongia, E. A. (2013). Effectiveness of monovalent 2009 pandemic influenza A virus subtype H1N1 and 2010-2011 trivalent inactivated influenza vaccines in Wisconsin during the 2010-2011 influenza season. The Journal of infectious diseases, 207(8), 1262–1269. https://doi.org/10.1093/infdis/jit020	Adults not isolated
97. Suzuki, M., Yoshimine, H., Harada, Y., Tsuchiya, N., Shimada, I., Ariyoshi, K., & Inoue, K. (2013). Estimating the influenza vaccine effectiveness against medically attended influenza in clinical settings: a hospital-based case-control study with a rapid diagnostic test in Japan. PloS one, 8(1), e52103. https://doi.org/10.1371/journal.pone.0052103	Insufficient information to determine outcomes
98. Centers for Disease Control and Prevention (CDC) (2013). Early estimates of seasonal influenza vaccine effectiveness--United States, January 2013. MMWR. Morbidity and mortality weekly report, 62(2), 32–35	Adults not isolated
99. Cheng, A. C., Brown, S., Waterer, G., Holmes, M., Senenayake, S., Friedman, N. D., Hewagama, S., Simpson, G., Wark, P., Upham, J., Korman, T., Dwyer, D., Wood-Baker, R., Irving, L., Bowler, S., Kotsimbos, T., & Kelly, P. (2013). Influenza epidemiology, vaccine coverage and vaccine effectiveness in sentinel Australian hospitals in 2012: the Influenza Complications Alert Network (FluCAN). Communicable diseases intelligence quarterly report, 37(3), E246–E252.	Adults not isolated

Table S2. Characteristics of the included RCT studies

(ILI: influenza like illness; ARI: Acute respiratory illness, n: sample size)

Author	Country	Season	Vaccine	Efficacy	Strain mismatch	Symptoms	Age range	Diagnostic tests
Liebowitiz 2020	USA	2012-2013	QIV	43%	Matched	ILI	18-65	PCR
Madhi 2014a	South Africa	2011-2012	TIV	50.4%	Mismatched	ARI	18-38	PCR
Madhi 2014b	South Africa	2011-2012	TIV	57.7%	Unclear	ILI	18-38	PCR
Macbride 2017a	Australia	2008-2009	TIV	60.10%	Matched	ILI	18-64	PCR
Macbride 2017b	Australia	2008-2009	TIV	42%	Unclear	ILI	18-64	PCR
Steinhoff 2017a	Nepal	2011-2012	TIV	19%	Matched	ILI	15-40	PCR
Steinhoff 2017b	Nepal	2012-2013	TIV	0%	Unclear	ILI	15-40	PCR
Petrie 2016a	USA	2007-2008	TIV	70%	Matched	ILI	18-49	PCR
Petrie 2016b	USA	2007-2008	LAIV	38 %	Matched	ILI	18-49	PCR

Table S3. Characteristics of the included TNS studies

(ILI: influenza like illness; ARI: Acute respiratory illness; RT: rapid test)

Author	Country	Season	Vaccine	Effectiveness (95% CI) extracted from the paper	Strain mismatch	Symptoms	Age range	Diagnostic tests
Kissling 2023	Europe	2021-2022	QIV and LAIV	41 (25-64)	Mismatched	ILI and ARI	15-64	PCR
Tenforde 2023	USA	2021-2022	QIV	29 (24-33)	Mismatched	ILI	18-64	PCR
Kim 2022	Canada	2021-2022	QIV	not available	Mismatched	ILI	20-64	PCR
Price 2022	USA	2021-2022	QIV	not available	Mismatched	ILI	18-49	PCR
Richard 2022a	USA	2012-2013	LAIV	not available	Mismatched	ILI	18-64	PCR, RT, culture
Richard 2022b	USA	2013-2014	LAIV	not available	Mismatched	ILI	18-64	PCR, RT, culture
Richard 2022c	USA	2014-2015	LAIV	not available	Mismatched	ILI	18-64	PCR, RT, culture
Richard 2022d	USA	2012-2013	TIV	not available	Mismatched	ILI	18-64	PCR, RT, culture
Richard 2022e	USA	2013-2014	TIV	not available	Mismatched	ILI	18-64	PCR, RT, culture
Richard 2022f	USA	2014-2015	TIV	not available	Mismatched	ILI	18-64	PCR, RT, culture
Sominina 2021	Russia	2018-2019	TIV	62 not adjusted	Matched	ARI	18-64	PCR
Hyder 2021a	India	2018-2019	QIV	24 (-66–66)	Mismatched	ILI	18-49	PCR
Hyder 2021b	India	2018-2019	QIV	49 (-78-85)	Mismatched	ILI	50-64	PCR
Sturmann 2021a	Europe	2019-2020	TIV and QIV	29 (-3-53)	Mismatched	ARI	18-64	PCR
Stuurman 2021b	Europe	2019-2020	TIV and QIV	30 (-8-71)	Mismatched	ILI	18-64	PCR
Grijalva 2021a	USA	2019-2020	TIV and QIV	62 (27-81)	Mismatched	ARI	18-49	PCR
Grijalva 2021b	USA	2019-2020	TIV and QIV	20 (-48-57)	Mismatched	ARI	50-64	PCR
Hu 2021	USA	2019-2020	Unclear	46 (36-55)	Mismatched	ILI	18-64	PCR
Martin 2020[a]	USA	2016-2017	QIV	31 (12.4-45)	Matched	ARI	>18	PCR
Martin 2020b	USA	2017-2018	QIV	34.2 (21.9-44.6)	Matched	ARI	>18	PCR
Stuurman 2020a	Europe	2018-2019	QIV	40 (2-63)	Matched	ARI and ILI	18-64	PCR and RT
Stuurman 2020b	Europe	2018-2019	QIV	45 (18-63)	Matched	ARI and ILI	18-64	PCR and RT
Rizzo 2020	Italy	2018-2019	QIV	40.5 (18.7-56.4)	Mismatched	ARI	18-64	PCR
Qahtami 2020	Saudi Arabia	2018-2019	TIV	42 (14-64)	Unclear	ILI	18 – working age	PCR
Redlberger-Fritz 2020a	Austria	2016-2017	QIV, TIV, aTIV,LAIV	-7 (-131-51)	Mismatched	ILI	15-64	PCR
Redlberger-Fritz 2020b	Austria	2017-2018	QIV, TIV, aTIV,LAIV	19 (-63-60)	Mismatched	ILI	15-64	PCR
Redlberger-Fritz 2020c	Austria	2018-2019	QIV, TIV, aTIV,LAIV	51 (-2-76)	Matched	ILI	15-64	PCR

Rose 2020	Europe	2019-2020	QIV, TIV and LAIV	36 (1-58)	Mismatched	ARI and ILI	18-64	PCR
Ando 2019	Japan	2018-2019	QIV	43.4 (17.3-61.2	Unclear	ILI	16-64	RT
Segaloff 2019a	USA	2014-2015	TIV	41.1 (1.7-64.7)	Mismatched	ARI	Adults	PCR
Segaloff 2019b	USA	2015-2016	TIV	68.7 (44.6-82.5)	Matched	ARI	Adults	PCR
Flannery 2019	USA	2018-2019	TIV and QIV	Not available	Mismatched	ILI	18-64	PCR
Kissling 2019	Europe	2016-2017	TIV	34 (18-46)	Mismatched	ILI	15-64	PCR
Blanchette 2019	Canada	2010-2011	TIV	34 (20-40)	Unclear	ARI	18-64	PCR
Constantino 2019	Italia	2018-2019	QIV and TIV	59.5 (0.03-83.1)	Matched	ILI	15-64	PCR
Pebody 2019	United Kingdom	2017-2018	QIV and TIV	12.2 (-16.8-34)	Unclear	ILI	15-64	PCR
Chon 2019	Japan	2015-2016	QIV	27.3 not adjusted	Unclear	ILI	19-64	PCR and RT
Molgaard-Nielsen 2019	Dinamarca	2010-2011	TIV	63.9 (29.1-81.6)	Unclear	ILI	Pregnant women	Unclear
Regan 2019	Australia	2016	QIV	31 (3-51)	Mismatched	ILI	18-64	PCR
Kissling 2019a	Dinamarca	2018-2019	QIV and TIV	55 (44-64)	Mismatched	ILI	18-64	PCR
Kissling 2019b	European Union	2018-2019	QIV and TIV	32 (-31-65)	Mismatched	ILI	18-64	PCR
Kissling 2019c	United Kingdom	2018-2019	QIV and TIV	37 (-20-67)	Mismatched	ILI	18-64	PCR
Kissling 2019d	Dinamarca	2018-2019	QIV and TIV	39 (14-67)	Mismatched	ILI	18-64	PCR
Showronski 2019	Canada	2017-2018	Unclear	63 (46-75)	Matched	ILI	20-64	PCR
Regan 2019a	Australia	2012	TIV	46% not adjusted	Unclear	ILI	18-64	PCR
Regan 2019b	Australia	2013	TIV	57% not adjusted	Unclear	ILI	18-64	PCR
Regan 2019c	Australia	2014	TIV	60% not adjusted	Unclear	ILI	18-64	PCR
Regan 2019d	Australia	2015	TIV	50% not adjusted	Unclear	ILI	18-64	PCR
Thompson 2018a	USA	2010-2011	Unclear	72 (-5-93)	Unclear	ARI	18-50	PCR
Thompson 2018b	USA	2011-2012	Unclear	47 (-98-86)	Unclear	ARI	18-50	PCR
Thompson 2018c	USA	2012-2013	Unclear	23 (-85-68)	Unclear	ARI	18-50	PCR
Thompson 2018d	USA	2013-2014	Unclear	51 (-30-82)	Unclear	ARI	18-50	PCR
Thompson 2018e	USA	2014-2015	Unclear	24 (-189-47)	Unclear	ARI	18-50	PCR
Thompson 2018f	USA	2015-2016	Unclear	40 (-33-72)	Unclear	ARI	18-50	PCR
Flannery 2018a	USA	2017-2018	QIV and TIV	19 (0-34)	Matched	ILI	18-49	PCR
Flannery 2018b	USA	2017-2018	QIV and TIV	40 (24-53)	Matched	ILI	50-64	PCR
Chan 2018	China	2017-2018	QIV and TIV	71 (42.7-85.8)	Unclear	ILI	18-64	PCR
Seki 2018	Japan	2016-2017	QIV	36.5%	Matched	ILI	15-65	RT
Wu 2018	China	2016-2017	TIV	4 (-284-76)	Mismatched	ILI	18-59	PCR
Yaron-Yakobi 2018a	Israel	2014-2015	QIV, TIV, LAIV	-53.7 (-116.8-91.4)	Mismatched	ILI	18-64	PCR
Yaron-Yakobi 2018b	Israel	2015-2016	QIV, TIV, LAIV	39.1 (7.8-59.8)	Mismatched	ILI	18-64	PCR
Showronski 2018	Canada	2017-2018	QIV and TIV	31 (6-49)	Unclear	ILI	20-64	PCR

Stein 2017a	Israel	2016-2017	QIV and TIV	12.5 (-108.7-63.7)	Matched	ILI	18-44	PCR
Stein 2017b	Israel	2016-2017	QIV and TIV	58.8 (.8-82.9)	Matched	ILI	45-64	PCR
Pelody 2017	United Kingdom	2017-2018	QIV and TIV	12.2 not adjusted	Mismatched	ILI	18-64	PCR
Showronski 2017a	Canada	2015-2016	TIV	not available	Matched	ILI	20-49	PCR
Showronski 2017b	Canada	2015-2016	TIV	not available	Matched	ILI	50-64	PCR
Kuliese 2017	Lithuania	2015-2016	TIV	61 (-345-97)	Unclear	ARI	18-64	PCR
Ma 2017	China	2014-2015	TIV	60 (-415-50)	Mismatched	ILI	18-59	PCR
Seki 2017a	Japan	2013-2014	QIV	56.3 (20.9-75.9)	Unclear	ILI	15-65	RT
Seki 2017b	Japan	2014-2015	QIV	7.7 (-64-48.1)	Unclear	ILI	15-65	RT
Seki 2017c	Japan	2015-2016	QIV	52.9 (20-72.3)	Matched	ILI	15-65	RT
McAnerney 2016	South Africa	2015	TIV	54.4 (-14.1-81.8)	Unclear	ILI	18-64	PCR
Fielding 2016	Australia	2015	TIV	52 (37-63)	Mismatched	ILI	18-64	PCR
Petrie 2016a	USA	2014-2015	QIV	67.4 (10.5-88.1)	Mismatched	ARI	18-49	PCR
Petrie 2016b	USA	2014-2015	QIV	9.7 (-126.5-64)	Mismatched	ARI	50-64	PCR
Rizzo 2016	Italy	2014-2015	TIV	-6.3 (-133.6-51.6)	Mismatched	ILI	15-64	PCR
Lytras 2016	Greece	2014-2015	TIV	not available	Mismatched	ARI	15-59	PCR
Rondy 2016	Europe	2013-2014	TIV	not available	Matched	ILI	18-64	PCR
Gherasim 2016	Spain	2014-2015	TIV	not available	Mismatched	ILI	15-64	PCR
Redlberger-Fritz 2016	Austria	2014-2015	TIV	54 (-13-82)	Mismatched	ILI	15-64	PCR
Kelly 2016a	Australia	2011	TIV	not available	Matched	ILI	18-64	PCR
Kelly 2016b	Australia	2011	TIV	not available	Matched	ILI	18-64	PCR
Kelly 2016c	Australia	2012	TIV	not available	Mismatched	ILI	18-64	PCR
Kelly 2016d	Australia	2012	TIV	not available	Mismatched	ILI	18-64	PCR
Kelly 2016e	Australia	2013	TIV	not available	Mismatched	ILI	18-64	PCR
Kelly 2016f	Australia	2013	TIV	not available	Mismatched	ILI	18-64	PCR
Bissielo 2016a	New Zeland	2015	TIV	27 (-8-51)	Mismatched	ILI	18-64	PCR
Bissielo 2016b	New Zeland	2015	TIV	46 (1-70)	Mismatched	ARI	19-64	PCR
Cheng 2015	Australia	2014	TIV	49.7 (35.3-60.8)	Matched	ARI	16-64	PCR
Levy 2015a	Thailand	2009-2010	TIV	not available	Mismatched	ILI	18-64	PCR
Levy 2015b	Thailand	2010-2011	TIV	not available	Matched	ILI	18-64	PCR
Levy 2015c	Thailand	2011-2012	TIV	not available	Matched	ILI	18-64	PCR
Levy 2015d	Thailand	2012-2013	TIV	not available	Mismatched	ILI	18-64	PCR
McLean 2015	USA	2012-2013	TIV	47	Matched	ARI	18-64	PCR
McAnerney 2015a	South Africa	2010	TIV	48.4 (-22.2-78.2)	Matched	ILI	18-64	PCR
McAnerney 2015b	South Africa	2011	TIV	58.7 (9.7-81.1)	Matched	ILI	18-64	PCR
McAnerney 2015c	South Africa	2012	TIV	67.1 (-15.6-90.6)	Mismatched	ILI	18-64	PCR

McAnerney 2015d	South Africa	2013	TIV	90.7 (36.8-98.6)	Matched	ILI	18-64	PCR
McAnerney 2015e	South Africa	2014	TIV	42.7 (-106.7-84.1)	Mismatched	ILI	18-64	PCR
Rondy 2015	Europe	2012-2013	TIV	not available	Mismatched	ILI	Adultos	PCR
Mclean 2015	USA	2012-2013	TIV	not available	Matched	ARI	18-64	PCR
Filipovic 2014	Croatia	2010-2011	TIV	-19.5 (-240.6-58.1) not adjusted	Matched	ILI	18-64	PCR
Turner 2014a	New Zeland	2014	TIV	not available	Unclear	ARI	18-64	PCR
Turner 2014b	New Zeland	2014	TIV	not available	Unclear	ILI	18-64	PCR
Levy 2014a	Australia	2010	TIV	60 (-1-84)	Matched	ILI	18-64	PCR
Levy 2014b	Australia	2011	TIV	40 (-22-70)	Matched	ILI	18-64	PCR
Levy 2014c	Australia	2012	TIV	47 (19-65)	Matched	ILI	18-64	PCR
Yang 2014	China	2012-2013	TIV	not available	Matched	ILI	18-59	Vírus isolation
Sullivan 2014	Australia	2012	TIV	12 (-22-36)	Mismatched	ILI	18-64	PCR
Skowronski 2014a	Canada	2011-2012	TIV	56 (26-74)	Mismatched	ILI	18-64	PCR
Skowronski 2014b	Canada	2013-2014	TIV	not available	Matched	ILI	18-64	PCR
Skowronski 2014c	Canada	2012-2013	TIV	not available	Mismatched	ILI	18-64	PCR
Kavanagh 2013	Scotland	2010-2011	TIV	100 (-349-100)	Matched	ILI	18-64	PCR
Castilla 2013	Spain	2011-2012	TIV	44 (-11-72)	Matched	ILI	15-59	PCR

Table S4. ROBINS-I overall assessment of the included TND studies by domain

(0: No information; 1: Low; 2: Moderate; 3: Serious; 4: Critical)

Author	Year	DOMAIN							
		Confounding bias	Selection bias	Classification	Deviation from intended intervations	Missing	Outcome	Selection of subgroup	Overall bias
Kissling et al	2023	1	4	0	1	1	1	1	0
Tenforde et al	2023	0	1	1	1	1	1	1	0
Kim et al	2022	4	1	4	1	1	1	1	4
Price et al	2022	4	1	3	1	1	1	1	4
Richard et al	2022	4	1	0	1	1	4	1	0
Sominina A. Et al	2021	4	1	4	1	1	1	1	4
Mir, Hyder et al	2021	1	1	3	1	1	1	1	3
Stuurmann, A et al	2021	1	1	3	1	1	1	1	3
Grijalva C.G. et al	2021	1	1	1	1	1	1	1	1
Hu, Wemping et al	2021	1	1	3	1	1	1	1	3
Martin E. T. et al	2020	1	1	2	1	1	4	1	4
Stuurman A. L. et al	2020	1	1	1	1	3	4	1	4
Rizzo C. et al	2020	1	4	1	1	1	1	1	4
Qahtami A.A.A. Et al	2020	1	1	1	1	1	1	1	1
Redlberger-Fritz M. et al	2020	1	1	0	1	1	1	1	0
Rose A. Et al	2020	1	1	0	1	1	1	1	0
Ando S.	2019	1	1	0	1	1	1	1	0
Segaloff H. et al	2019	1	1	4	1	1	1	1	4
Flannery B. et al	2019	4	1	4	1	1	1	1	4
Kissling E. et al	2019	1	1	4	1	3	1	1	4
Blanchette, p et al	2019	1	1	1	1	1	1	1	1
Constantino C. Et al	2019	1	1	1	1	1	1	1	1
Pebody R et al	2019	1	1	4	1	1	1	1	4
Chon I. et al	2019	4	1	0	1	1	4	1	0
Molgaard-Nielsen et al	2019	1	1	1	1	3	4	1	4
Regan A et al	2019	1	1	1	1	1	1	1	1
Kissling E. et al	2019	1	1	0	1	0	1	1	0
Showronski D et al	2019	1	1	4	1	1	1	1	4
Regan A. Et al	2019	1	1	1	1	1	1	1	1

Thompson M. et al	2018	1	1	0	1	1	0	1	0
Flannery B. et al	2018	1	1	3	1	1	1	1	3
Chan Y. et al	2018	1	1	4	1	1	4	1	4
Seki Y. et al	2018	1	1	0	1	1	1	1	0
Wu S. et al	2018	1	1	0	1	1	1	1	0
Yaron-Yakobi H. et al	2018	1	1	4	1	1	1	1	4
Showronski D et al	2018	1	1	3	1	1	1	1	3
Stein Y. et al	2017	1	1	4	1	1	1	1	4
Pelody R. et al	2017	4	1	4	1	1	1	1	4
Showronski D et al	2017	4	1	4	1	1	1	1	4
Kuliese M. et al	2017	1	1	1	1	3	1	1	3
Ma C. et al	2017	1	1	1	1	1	1	1	1
Seki Y. et al	2017	1	1	1	1	1	1	1	1
McAnerney J. et al	2016	1	1	3	1	1	1	1	3
Fielding J. et al	2016	1	1	3	1	1	1	1	3
Petrie J. et al	2016	1	1	4	1	1	1	1	4
Rizzo C. et al	2016	1	1	4	1	1	1	1	4
Lytras t. et al	2016	4	1	4	1	1	1	1	4
Rondy A. Et al	2016	4	1	3	1	1	1	1	4
Gherasim A. Et al	2016	4	4	3	1	1	1	1	4
Redlberger-Fritz M. et al	2016	1	1	0	1	1	1	1	0
Kelly H. et al	2016	4	1	3	1	1	1	1	4
Bissielo A. et al	2016	1	1	1	1	1	1	1	1
Cheng et al	2015	1	1	3	1	4	1	1	4
Levy et al	2015	4	1	4	1	1	1	1	4
McAnerney et al	2015	1	1	3	1	1	1	1	3
McAnerney et al	2015	1	1	3	1	1	1	1	3
Rondy et al	2015	4	1	3	1	1	1	1	4
Filipovic et al	2015	4	3	3	1	1	1	1	4
McLean et al	2015	4	1	1	1	1	1	1	4
Turner et al	2014	4	1	3	1	1	1	1	4
Levy et al	2014	1	1	3	1	1	1	1	3
Yang et al	2014	4	1	4	1	1	1	1	4
Sullivan et al	2014	1	1	0	1	1	1	1	0
Skowronski et al	2014	4	1	4	1	1	4	1	4

Skowronski et al	2014	4	1	0	1	1	1	1	0
Skowronski et al	2014	2	1	4	1	1	1	1	4
Kavanagh et al	2013	1	1	1	1	1	1	1	1
Castilla et al	2013	1	1	1	1	1	1	1	1
